# Development and field evaluation of PCR assays based on minimum length *Bm86* cDNA fragments required for *Rhipicephalus* and *Hyalomma* tick species delineation

**DOI:** 10.3389/fvets.2023.1209210

**Published:** 2023-06-29

**Authors:** Sayed Zamiti, Moez Mhadhbi, Mokhtar Dhibi, Mohamed Aziz Darghouth, Mourad Ben Said

**Affiliations:** ^1^Laboratory of Parasitology, National School of Veterinary Medicine of Sidi Thabet, University of Manouba, Manouba, Tunisia; ^2^Department of Basic Sciences, Higher Institute of Biotechnology of Sidi Thabet, University of Manouba, Manouba, Tunisia

**Keywords:** *in silico* selection, minimum length partial sequences, *Bm86* cDNA, *Hyalomma and Rhipicephalus* spp., species delineation

## Abstract

**Introduction:**

*Hyalomma* and *Rhipicephalus* ticks are important genera that can transmit diseases to both animals and humans, including Crimean-Congo hemorrhagic fever, tick-borne encephalitis, and several types of spotted fever. The accurate identification of tick species is essential for the effective control and prevention of tick-borne diseases. However, traditional identification methods based on morphology can be challenging and subjective, leading to errors. The development of DNA markers has provided more precise and efficient methods for tick species identification, but the currently available markers have limitations in their discriminatory power and sensitivity. To address this need for more sensitive and specific markers, this study aimed to identify two minimum sequence fragments required for tick *Hyalomma* and *Rhipicephalus* species identification using the *Bm86* cDNA marker, which has previously been shown to be in perfect agreement with the current taxonomy of hard ticks based on its complete sequence.

**Methods:**

Based on our *in silico* determination that a minimum sequence of 398 bp for *Rhipicephalus* spp. (from 1487 to 1884) and 559 bp for *Hyalomma* species (from 539 to 1097) was necessary for species delineation, two distinct PCR assays were developed to apply these sequences in practice.

**Results and discussion:**

Discrimination between species within each genus was achieved through sequence homology and phylogenetic analysis following the sequencing of the two PCR products. Subsequently, their performance was evaluated by testing them on the field-collected ticks of the *Hyalomma* and *Rhipicephalus* genera obtained from various host animals in different geographic regions of Tunisia. The use of shorter partial sequences specific to the tick genera *Rhipicephalus* and *Hyalomma*, which target the tick's RNA banks, could represent a significant advance in the field of tick species identification, providing a sensitive and discriminatory tool for interspecific and intraspecific diversity analysis.

## 1. Introduction

*Rhipicephalus* is a genus of ticks that includes several species known to transmit a range of pathogens to both animals and humans (1). Some of the most common species of *Rhipicephalus* ticks include *R. sanguineus* sensu lato, *R. microplus*, and *R. annulatus* (2). These ticks are distributed in different regions worldwide, with *R. sanguineus* s.l. found in the temperate and tropical regions and *R. microplus* and *R. annulatus* being more commonly found in tropical and subtropical regions (3). *Rhipicephalus* ticks are known to transmit various pathogens, including bacteria, viruses, and protozoans, such as Crimean-Congo hemorrhagic fever virus, *Babesia, Anaplasma, Ehrlichia*, and *Rickettsia* species, which can cause diseases such as Crimean-Congo hemorrhagic fever, Lyme disease, Rocky Mountain spotted fever, and other tick-borne illnesses (3, 4).

The genus *Hyalomma* comprises several species of hard ticks that are widely distributed in Africa, Asia, and Europe (5). These ticks are known to transmit a number of pathogens, including viruses, bacteria, and protozoa, some of which can cause severe diseases in humans and animals (3). *Hyalomma* ticks are important vectors of the Crimean-Congo hemorrhagic fever virus, a highly pathogenic virus that can cause severe hemorrhagic fever in humans (6, 7). They are also known to transmit several other viral pathogens, such as the Nairobi sheep disease virus and the Alkhumra virus (8). In addition, *Hyalomma* ticks can transmit bacterial pathogens such as *Rickettsia, Ehrlichia*, and *Anaplasma*, as well as protozoan pathogens such as *Theileria* and *Babesia* (9–12). Some *Hyalomma* species are also associated with the transmission of tick-borne encephalitis virus, a flavivirus that causes a range of neurological symptoms in humans (13).

The identification of *Rhipicephalus* and *Hyalomma* species using morphological diagnosis keys might be constrained by several factors (14). For instance, species from the *R. sanguineus* group have very similar morphologies, making their distinction quite laborious (15) and requiring therefore advanced taxonomic knowledge (i.e., male sclerites) for reliable species identification. In addition, tick species morphological diagnosis could be rendered problematic due to the alteration of some key morphological traits during the course of engorgement, in particular, for female ticks. Moreover, some species may exhibit significant variations in morphology depending on their geographic location or the host they are feeding on (16). Finally, in comparison to adult ticks, species identification is more problematic with juvenile stages of *Rhipicephalus* and *Hyalomma* due to the absence of expression of some key morphological features present in adults. Accordingly, morphological diagnosis can be time-consuming, requiring specialized knowledge and training, and these taxonomic competences may not be readily available in some regions (17). These challenges underscore the importance of incorporating additional approaches to morphological diagnosis, including molecular and genetic techniques, to identify and classify tick species more accurately (17).

The use of DNA markers has significantly improved the reliability of the identification of tick species, including those of the genera *Hyalomma* and *Rhipicephalus* (18). Compared to morphological diagnosis, DNA-based identification was shown, for several tick species, to be highly accurate and able to differentiate between closely related species (19). Some of the DNA markers used for species identification in these genera include mitochondrial DNA (mtDNA) genes, such as cytochrome c oxidase subunit 1 (CO1) and 16S rRNA, as well as nuclear DNA markers like the internal transcribed spacer (ITS) region (20–22). These markers were successfully applied to species identification for instance with *Hyalomma dromedarii* and *Rhipicephalus microplus* (23–25).

However, and despite their usefulness, there are limitations to the use of existing DNA markers for tick species identification (26, 27). Indeed, there are still gaps in the available DNA sequence databases for some tick species, emphasizing accordingly, the need to design more reliable molecular markers for their identification (28). Moreover, the existence of hybridization and introgression events between different tick species can further complicate the identification process. Therefore, the development of more comprehensive DNA sequence databases and the use of multiple molecular markers can help to increase the accuracy and reliability of tick species identification (29).

In this context, the present study targeted an mRNA-based marker as a complementary tool to morphological diagnosis and to genomic and/or mitochondrial DNA markers. Unlike genomic DNA markers, which have several limitations, mRNA markers provide more specific and sensitive information for species identification. mRNA is a complementary copy of a gene that is used as a template for protein synthesis (30). mRNA-based markers are highly conserved among species and are often more species-specific than genomic markers (31, 32). These markers can be used in combination with traditional morphological methods and other molecular markers to improve the accuracy of tick species identification.

Our study focused on identifying and validating mRNA-based markers for the diagnosis of *Hyalomma* and *Rhipicephalus* species. The rational base of our study was to determine the minimum length of partial *Bm86* cDNA fragments required for species identification in two tick genera, namely *Rhipicephalus* and *Hyalomma*. The decision to use *Bm86* cDNA encoding the vaccine target gut protein is justified by the fact that the complete cDNA sequence perfectly aligns with the recent classification of hard ticks (31). This strategy offers several expected benefits, including the potential to detect several tick species using a single molecular marker (33). Although changes in climate and in the length of the different seasons will directly affect tick survival, activity, and development, our mRNA-based markers can be applied to both engorged and non-engorged ticks, making it useful for analyzing ticks in various developmental stages and environmental settings (34, 35).

Therefore, the aims of this study were to conduct an *in silico* analysis to identify the minimum partial *Bm86* cDNA sequence required for the identification of species within the *Hyalomma* and *Rhipicephalus* genera, to develop a molecular method based on the amplification and sequencing of these two minimal sequences, and to apply this technique for the identification of tick field specimens belonging to the two genera collected from several hosts located in different Tunisian regions.

## 2. Materials and methods

### 2.1. Sequence and data acquisition from GenBank

In this study, a selection of minimum length partial *Bm86* cDNA fragments required for species delineation within *Rhipicephalus* and *Hyalomma* tick genera was performed. This selection was based on all complete or nearly complete *Bm86* cDNA sequences available in GenBank on 1 January 2023. All sequences were screened and downloaded from GenBank (http://www.ncbi.nlm.nih.gov/GenBank/) through a BLAST analysis (maximum and minimum recovery of 100 and 68%, respectively). In particular, 175 isolates or strains belonging to the six classified *Rhipicephalus* species and 19 isolates or strains belonging to the five classified *Hyalomma* species, which is available in the GenBank^®^ database of NCBI (http://www.ncbi.nlm.nih.gov/nuccore/), were analyzed in this study (36–45) (Supplementary Tables 1–3).

### 2.2. Multiple sequence alignments

**Figure 1:**
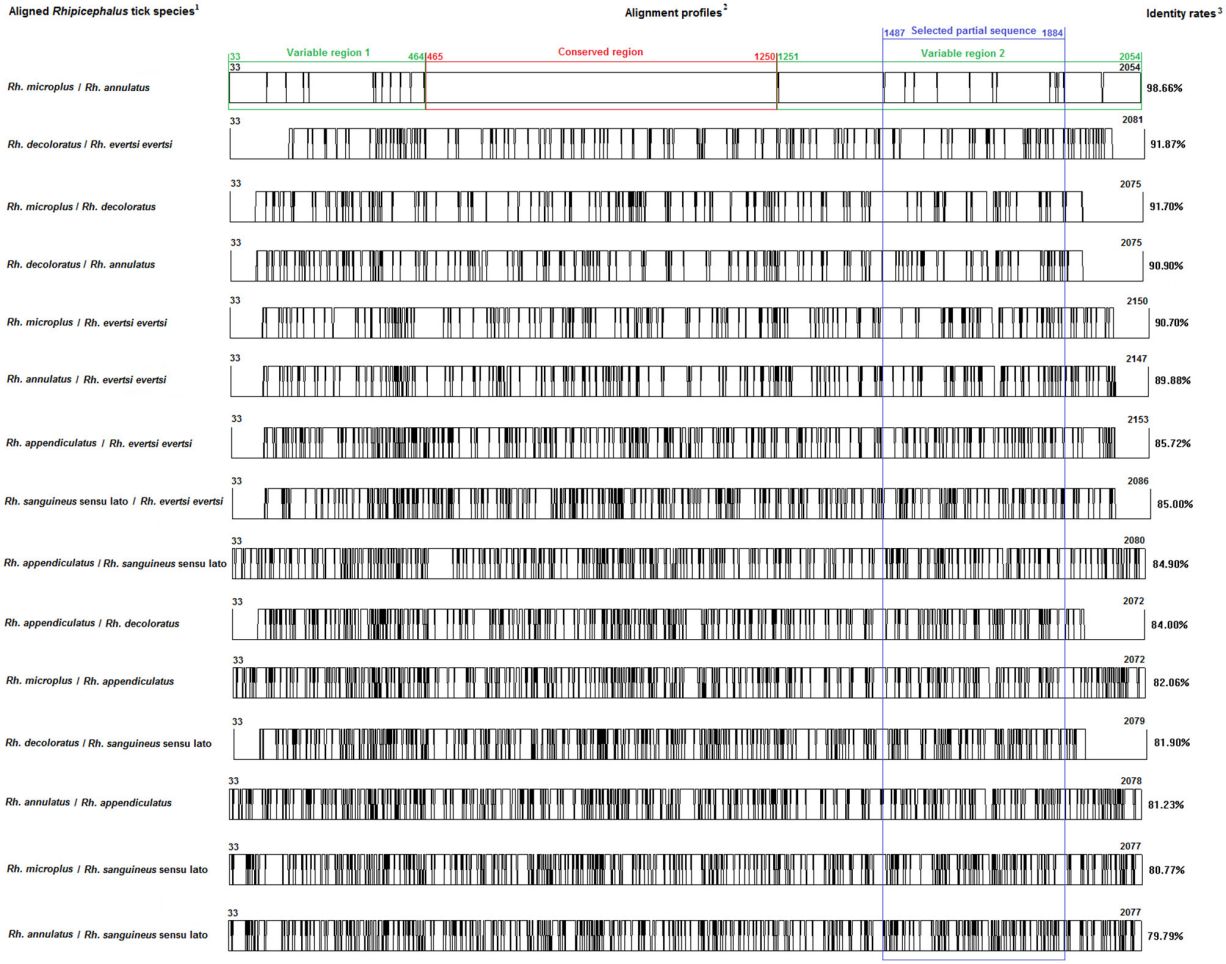
Multiple alignment profiles allowing the selection of minimum length *Bm86* cDNA fragments required for species delineation within *Rhipicephalus* tick genera. ^1^*Rhipicephalus* species analyzed in each alignment profile. ^2^Alignment profiles of all available genetic variants isolated from each *Rhipicephalus* species. ^3^Average percent identity between genetic variants of each *Rhipicephalus* species.

**Figure 2:**
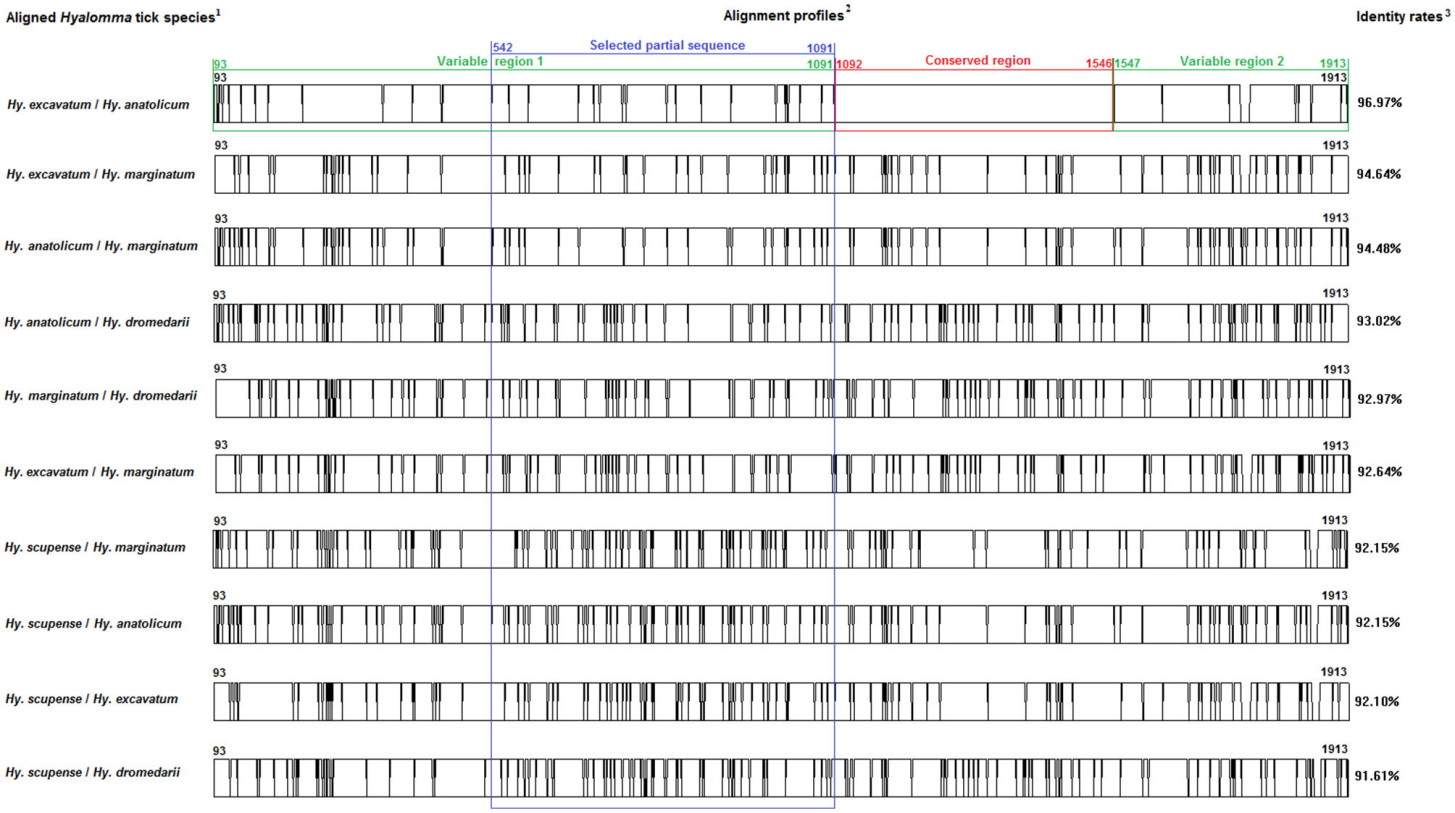
Multiple alignment profiles allowing the selection of minimum length *Bm86* cDNA fragments required for species delineation within *Hyalomma* tick genera. ^1^*Hyalomma* species analyzed in each alignment profile. ^2^Alignment profiles of all available genetic variants isolated from each *Hyalomma* species. ^3^Average percent identity between genetic variants of each *Hyalomma* species.

Multiple sequence alignments and sequence similarities were calculated using the CLUSTAL W 1.81. The DNAMAN software (Version 5.2.2; Lynnon Biosoft, Que., Canada) was also used to create two *Bm86* sequences' alignment profiles. Partial *Bm86* sequences that ensure effective discrimination between species of *Rhipicephalus* and *Hyalomma* genera were selected to investigate interspecific diversity positions with the analysis of sequence alignments (Figures 1, 2).

### 2.3. Collection and species identification of tick samples

Between June and August 2020, adult tick specimens were collected from four different bioclimatic areas and five governorates (Table 1). Specimens were hand-picked from live domestic animals (e.g., cattle, camels, goats, sheep, and dogs). Tick specimens were identified based on morphological characteristics according to the identification keys of Walker et al. (46). Tick samples were preserved in 1 ml of TRIzol reagent (Invitrogen life technologies, Invitrogen Corporation, California, USA) and stored at −80°C for subsequent uses.

**Table 1:** Information about origins and hosts of studied field tick specimens belonging to *Hyalomma* and *Rhipicephalus* genera from Tunisia and blast analysis of sequenced mitochondrial 16S rRNA and *Bm86* cDNA fragments.

**Tick specimen**	**Host species**	**District (governorate, bioclimatic area)**	**GenBank Acc. Nb. (mito16SrRNA/*Bm86*)**	**Morphological diagnosis**	**Blast analysis**	
					**Mito 16S rRNA partial sequence**	***Bm86*** **cDNA minimal sequence**
Hs1	*B. taurus*	Raoued (A, SA)	OP749949/OP762544	*Hy. scupense*	100% to *Hy. scupense* (MK601705)	100% to *Hy. scupense* (HQ872022)
Ha1	*B. taurus*	Amdoun (B, H)	OP749950/OP762545	*Hy. anatolicum*	100% to *Hy. anatolicum* (MT509435)	99.28% to *Hy. anatolicum* (EU665682)
He1	*B. taurus*	Nefza (B, H)	OP749951/OP762546	*Hy. excavatum*	100% to *Hy. excavatum* (MK601704)	99.10% to *Hy. excavatum* (JF298786)
He2	*B. taurus*	Kalâa Kebira (S, SA)	OP749952/OP762547	*Hy. excavatum*	100% to *Hy. excavatum* MK601704	99.11% to *Hy. excavatum* (JF298786)
Hm1	*B. taurus*	Raoued (A, SA)	OP749953/OP762548	*Hy. marginatum*	100% to *Hy. marginatum* (MT229186)	100% to *Hy. marginatum* (KF527438)
Hm2	*B. taurus*	Nefza (B, H)	OP749954/OP762549	*Hy. marginatum*	100% to *Hy. marginatum* (MT229186)	100% to *Hy. marginatum* (KF527438)
Hm3	*B. taurus*	El Fahs (Z, SA)	OP749955/OP762550	*Hy. marginatum*	100% to *Hy. marginatum* (MT229186)	100% to *Hy. marginatum* (KF527438)
Hi1	*C. dromedarius*	Remada (T, A)	OP749956/OP762551	*Hy. impeltatum*	100% to *Hy. impeltatum* (MN960583)	97.67% to *Hy*.*anatolicum*^1^ (MH325952)
Hd1	*C. dromedarius*	Remada (T, A)	OP749957/OP762552	*Hy. dromedarii*	100% to *Hy. dromedarii* (MN960589)	99.82% to *Hy. dromedarii* (JF298785)
Rb1	*O. aries*	Raoued (A, SA)	OP749958/OP762553	*Rh. bursa*	100% to *Rh. bursa* (MT302761)	96.61% to *Rh*.*evertsievertsi*^2^ (GU144600)
Rs1	*B. taurus*	Amdoun (B, H)	OP749959/OP762554	*Rh. sanguineus* s.l.	100% to *Rh. sanguineus* s.l. (MK732012)	97.34% to *Rh. sanguineus* s.l. (EF222203)
Rs2	*B. taurus*	Raoued (A, SA)	OP749960/OP762555	*Rh. sanguineus* s.l.	100% to *Rh. sanguineus* s.l. (KY413785)	97.34% to *Rh. sanguineus* s.l. (EF222203)
Rs3	*B. taurus*	Amdoun (B, H)	OP749961/OP762556	*Rh. sanguineus* s.l.	99.27% to *Rh. sanguineus* s.l. (MH630344)	96.85% to *Rh. sanguineus* s.l. (EF222203)
Rs4	*C. l. familiaris*	Sidi Thabet (A, SA)	OP749962/OP762557	*Rh. sanguineus* s.l.	100% to *Rh. sanguineus* s.l. (KY413785)	96.85% to *Rh. sanguineus* s.l. (EF222203)

*B. taurus* , *Bos taurus*; *C. dromedarius* , *Camelus dromedarius*; *O. aries* , *Ovis aries*; *C. l. familiaris* , *Canis lupus familiaris*; A , Ariana; B , Beja; S , Sousse; Z , Zaghouan; T , Tataouine; SA , Semi-arid; H , Humid; A , Arid; *Hy. scupense, Hyalomma scupense*; *Hy. anatolicum, Hyalomma anatolicum*; *Hy. excavatum, Hyalomma excavatum*; *Hy. marginatum, Hyalomma marginatum*; *Hy. impeltatum, Hyalomma impeltatum*; *Hy. dromedarii, Hyalomma dromedarii*; *Rh. bursa, Rhipicephalus bursa*; *Rh. sanguineus*; s.l., *Rhipicephalus sanguineus* sensu lato.

^1^ Since this is the first time that the *Bm86* ortholog has been sequenced in *Hyalomma impeltatum*, blast analysis indicated that the closest sequence was that of *Hyalomma anatolicum* from Iran (MH325952); ^2^ Since this is the first time that the *Bm86* ortholog has been sequenced in *Rhipicephalus bursa*, blast analysis indicated that the closest sequence was that of *Rhipicephalus evertsi evertsi* from South Africa (GU144600).

### 2.4. Total RNA and genomic DNA co-extraction

A total of 14 field tick specimens belonging to *Rhipicephalus* and *Hyalomma* genera were selected for the field evaluation of minimum length *Bm86* cDNA fragments. DNA and RNA co-extraction from a single adult tick specimen were performed using TRIzol reagent (Invitrogen, CA, USA). Each whole tick was crashed and homogenized in TRIzol reagent (Invitrogen, CA, USA) (1 mL/100 mg tissue) using a pestle and liquid nitrogen. Phases' separation was performed by adding chloroform in a TRIzol to chloroform ratio of 3:1 according to the manufacturer's instructions. For each sample, the aqueous phase was separated from the interphase/organic phase and subjected to RNA isolation according to the TRIzol reagent protocol. The final resuspension of total RNA was performed in 50 μl of RNase-free water, followed by incubation at 56°C for 10 min for complete resolubilization of the nucleic acid. RNA samples were immediately stored at −80°C until further use. The DNA samples present in the interphase/organic phase were purified according to the TRIzol reagent according to the manufacturer's instructions. DNA isolation was carried out with a final resuspension in 0.1–0.2 ml of 8 mM NaOH. Insoluble materials were pelleted by centrifugation for 10 min at 12,000 × g at 4°C. The supernatant was transferred to a new 1.5 ml tube and stored at −20°C until use.

### 2.5. Amplification of mitochondrial 16s rRNA partial sequences

To confirm the results of the morphological diagnosis, a partial mitochondrial 16S rRNA sequence (273 bp) was amplified from all analyzed tick specimens by using primers TQ16S+1F (5′-CTGCTCAATGATTTTTTAAATTGCTGTGG-3′) and TQ16S-2R (5′-ACGCTGTTATCCCTAGAG−3′), as previously described by Black and Piesman (47).

### 2.6. cDNA synthesis and amplification of *Bm86* partial sequences

The first-strand synthesis reaction was carried out using the SuperScript First-strand Synthesis System for the RT-PCR kit (Invitrogen USA) following the manufacturer's instructions. Two primer sets employed to amplify the selected minimum length partial *Bm86* cDNA fragments needed for species delineation within *Rhipicephalus* and *Hyalomma* tick genera were designed, and the position of each primer was obtained with respect to the *Bm86* cDNA sequence isolated from the *R. (B.) australis* (formerly *R. microplus*) Yeerongpilly strain (GenBank Accession number M29321) and the *Ha98* cDNA sequence isolated from the *H. anatolicum* India strain (GenBank accession number AF347079), for *Rhipicephalus* and *Hyalomma* tick genera, respectively (Table 2).

**Table 2:** Primers employed to amplify and sequence *Bm86* cDNA minimal sequence used for species delineation within *Hyalomma* and *Rhipicephalus* genera.

**Primer name**	**Nucleotide sequence (5'-3')**	**Primer position^1^**	**Amplified (sequenced)**^**2**^ **fragments**		***Bm86*** **cDNA minimal sequence**		**Annealing temperature (°C)**	**Reference**

			**Position**	**Size (bp)**	**Position**	**Size (bp)**		
AD_Bm86_Hyl	GCGAGAAAAACYTGCTTGGAAA	517-538	517-1119	603 (559)	539-1097	559	54	Present study
AR_Bm86_Hyl	TCTCRTACCACTCGCAATGGTC	1098-1119	(539-1097)					
AD_Bm86_Rhip	TTCTGGTTCCAGTGCGCTGA	1416-1435	1416-1917	502 (460)	1487-1884	398	57	Present study
AR_Bm86_Rhip	CAGCACTYGACTTYCCASGAT	1897-1917	(1436-1896)					

^1^Numbers represent the nucleotide position of AD_Bm86_Hyl/AR_Bm86_Hyl and AD_Bm86_Rhip/AR_Bm86_Rhip primer sets with respect to the India strain of *Hyalomma anatolicum* tick from India (GenBank accession number AF347079) (48) and the Yeerongpilly strain of *Rhipicephalus* (*Boophilus*) *microplus* tick from Australia (GenBank accession number M29321) (49).^2^Represent the amplified sequences without the primers' sequences.

**Figure 3:**
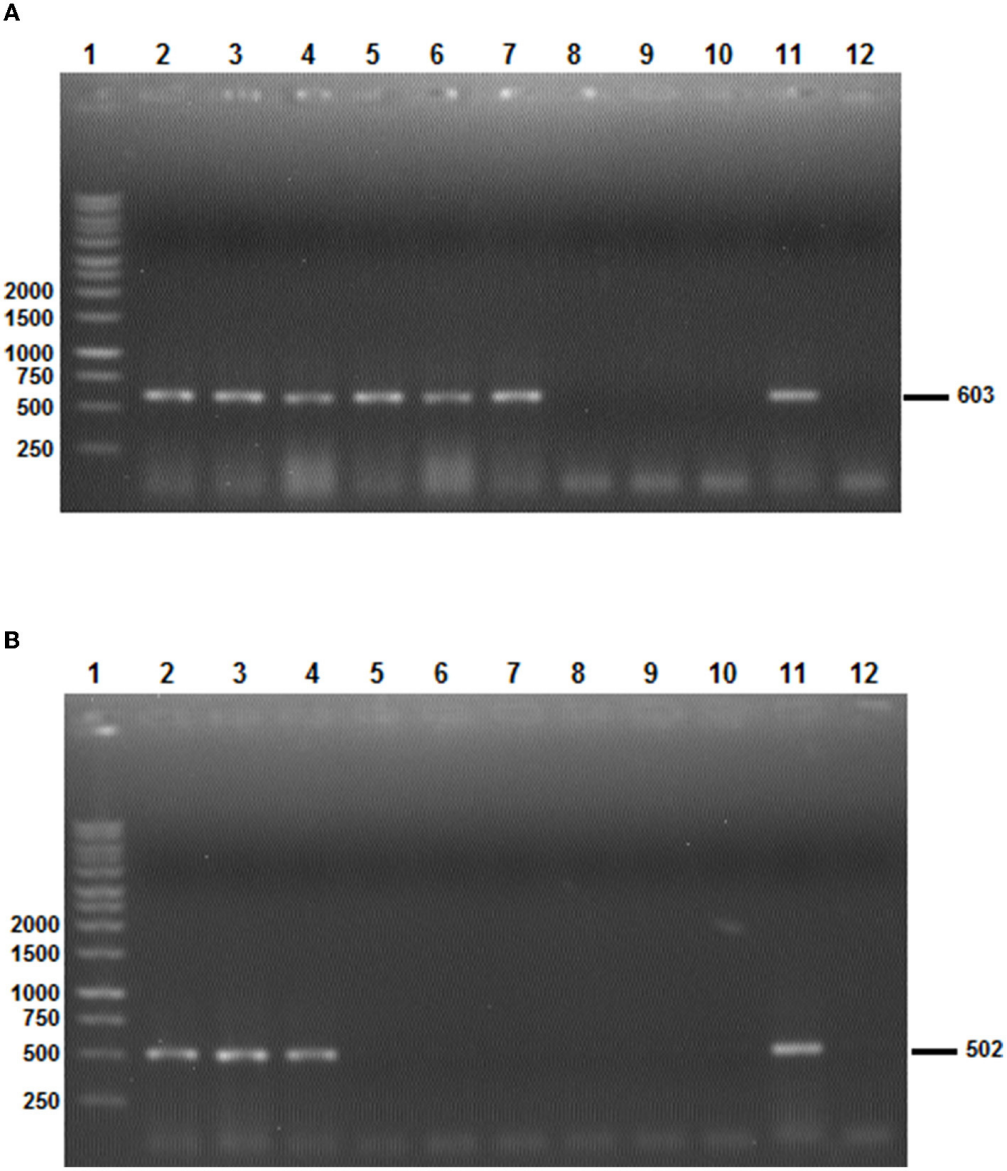
The results of the two *Bm86* minimal partial fragments' amplification specific to *Hyalomma* **(A)** and *Rhipicephalus* **(B)** species. **(A)** Line 1: 1 Kb ladder; lines 2–7: PCR products after the amplification of the *Hyalomma* spp. partial fragment based on *Bm86* cDNA orthologs isolated from *Hy. scupense, Hy. marginatum, Hy. excavatum, Hy. anatolicum, Hy. Dromedarii*, and *Hy. impeltatum*, respectively; lines 8–10: PCR products showing the absence of amplification of the *Hyalomma* spp. partial fragment based on *Bm86* cDNA orthologs isolated from *Rh. bursa, Rh. sanguineus* s.l (Rs1 from cattle), and *Rh. sanguineus* s.l (Rs4 from a dog), respectively; and lines 11 and 12: positive and negative controls, respectively. **(B)** Line 1: 1 Kb ladder; lines 2–4: PCR products after the amplification of *Rhipicephalus* spp. partial fragment based on *Bm86* cDNA orthologs isolated from *Rh. bursa, Rh. sanguineus* s.l (Rs1 from cattle), and *Rh. sanguineus* s.l (Rs4 from a dog); lines 5–10: PCR products showing the absence of amplification of the *Rhipicephalus* spp. partial fragment based on *Bm86* cDNA orthologs isolated from *Hy. scupense, Hy. marginatum, Hy. excavatum, Hy. anatolicum, Hy. Dromedarii*, and *Hy. impeltatum*, respectively; and lines 11 and 12: positive and negative controls, respectively.

All PCRs were performed in a final volume of 50 μl containing 0.125 U/μl of Taq DNA polymerase, 1 × PCR buffer 10 × , 2.5 mM of MgCl2, 0.2 mM of dNTPs, 4 μl (50 to 150 ng) cDNA, and 0.5 μM of primers. PCR reactions were performed in an automated DNA thermal cycler. The selected minimum length partial *Bm86* cDNA fragment (603 bp) for *Hyalomma* species delineation was amplified using the following conditions: an initial denaturation step of 5 min at 94°C followed by 40 cycles of 30 s at 94°C, 30 s at 54°C, 50 s at 72°C, and a final extension step of 72°C for 10 min. However, the thermal profile used for the amplification of partial *Bm86* cDNA fragment required for the *Rhipicephalus* spp. discrimination was as follows: an initial denaturation step of 5 min at 94°C followed by 35 cycles of 30 s at 94°C, 30 s at 57°C, 1 min at 72°C, and a final extension step of 72°C for 15 min. PCR products were observed after electrophoretic migration on 1% agarose gels stained with ethidium bromide and under UV transillumination (Figure 3).

### 2.7. Specificity and sensitivity of PCR assays

**Figure 4:**
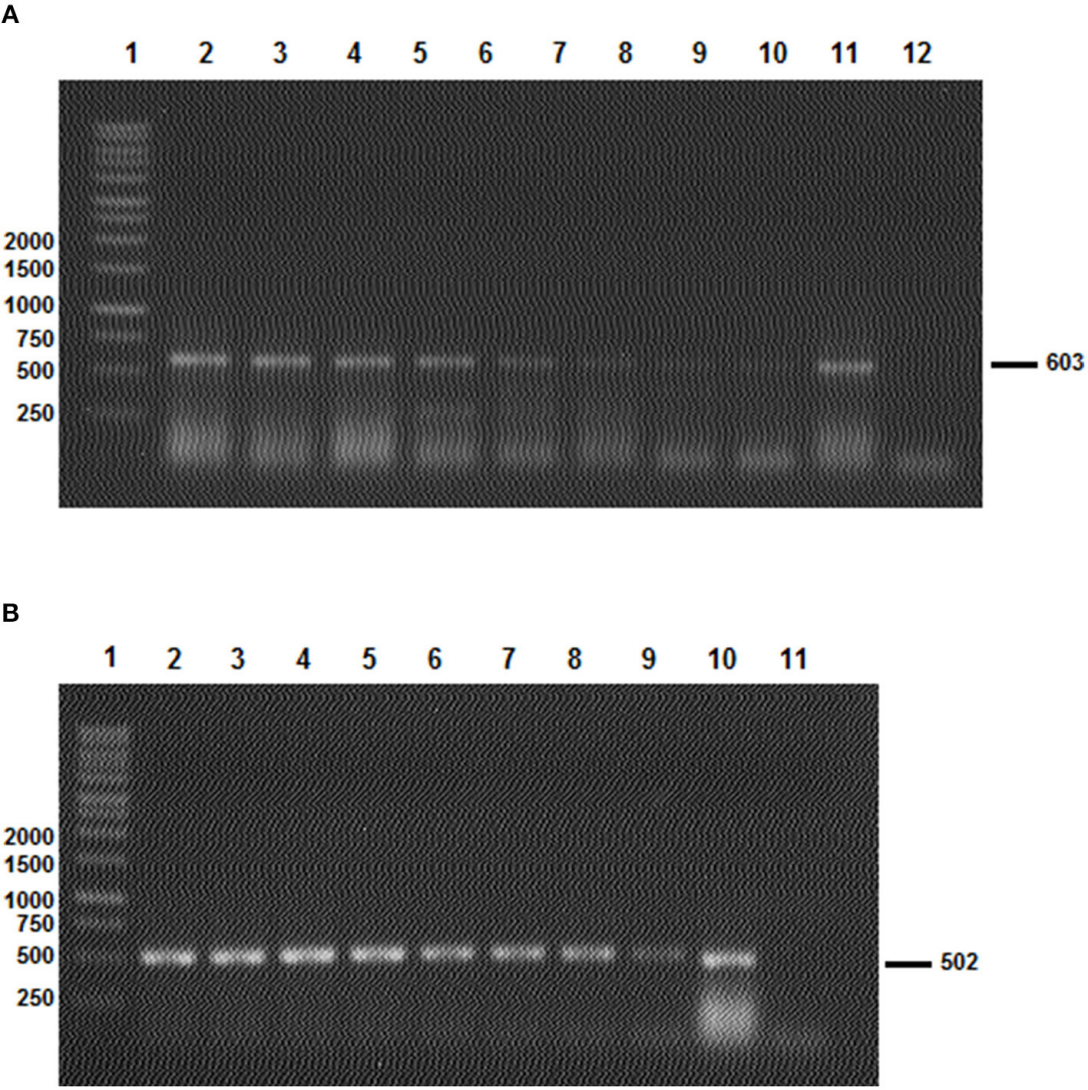
The results of the two *Bm86* minimal partial fragments' amplification sensibility test to *Hyalomma* **(A)** and *Rhipicephalus* **(B)** species. **(A)** Line 1: 1 Kb ladder; lines 2–10: PCR products after the amplification of the *Hyalomma* spp. partial fragment based on a serial dilution of a quantified *Bm86* cDNA (from 1.4 μg/μl to 1.4 × 10^−8^ μg/μl) isolated from *Hy. scupense* and lines 11 and 12: positive and negative controls, respectively. **(B)** Line 1: 1 Kb ladder; lines 2–4: PCR products after the amplification of *Rhipicephalus* spp. partial fragment based on a serial dilution of a quantified *Bm86* cDNA (from 1.9 μg/μl to 1.9 × 10^−8^ μg/μl) isolated from *Rh. sanguineus* s.l (Rs1 from cattle) and lines 10 and 11: positive and negative controls, respectively.

To determine the specificity of the PCR reactions, we utilized two sets of primers—AD-Bm86-Hyl/AR-Bm86-Hyl and AD-Bm86-Rhip/AR-Bm86-Rhip—to analyze samples of *Rhipicephalus* and *Hyalomma* spp. through PCR. In addition, to evaluate the sensitivity of the assay, we conducted serial dilutions of a quantified cDNA and calculated the PCR sensitivity in terms of the amount of cDNA per μl (Figure 4).

### 2.8. DNA sequencing, sequence analysis, and phylogenetic trees' construction

PCR products generated from mitochondrial 16S rRNA and *Bm86* partial sequences were purified and sequenced on both forward and reverse strands by using the same primers employed in PCR to obtain maximal data accuracy. The Big Dye Terminator cycle sequencing ready reaction kit (Applied Biosystems, Foster City, USA) and an ABI3730XL automated DNA sequencer (Macrogen Europe, Amsterdam, The Netherlands) were employed.

The chromatograms were evaluated with Chromas Lite v 2.01 (http://www.technelysium.com.au/chromas_lite.html). Obtained raw sequences were edited, primer region sequences were manually removed, and the overlapping parts were selected. BLAST analysis was performed to assess the level of similarity with previously reported sequences (http://blast.ncbi.nlm.nih.gov/).

Nucleotide sequences of the partial *Bm86* orthologs were compared with previously reported sequences isolated from *Hyalomma* and *Rhipicephalus* spp. ticks using the DNAMAN program (Version 5.2.2; Lynnon Biosoft, Quebec, Canada). By using the same software, genetic distances were computed by the maximum composite likelihood method (50) and were used to construct neighbor-joining trees (50). Statistical support for the internal branches of trees was established by bootstrap analysis with 1,000 reiterations (51).

The mitochondrial 16S rRNA and *Bm86* partial sequences of *Hyalomma* and *Rhipicephalus* spp. isolates were deposited in the GenBank under the accession numbers OP749949-OP749962 and OP762544-OP762557, respectively.

## 3. Results

### 3.1. Selection of minimum length partial *Bm86* cDNA fragments needed for species delineation

#### 3.1.1. Identification of genetic variants

Based on all available complete and nearly complete sequences of *Bm86* cDNA isolated from *Rhipicephalus* and *Hyalomma* species, we precisely selected the minimal length partial sequences that will allow us to discriminate between all classified species within each tick genus. All genetic variants of *Bm86* cDNA belonging to each *Rhipicephalus* and *Hyalomma* species were included in our data set. These genetic variants differ from each other by at least one mutation and represent complete or nearly complete sequences of all strains and isolates submitted in GenBank (Supplementary Tables 1–3). For all available *Rhipicephalus* and *Hyalomma* species, the two genetic variants (each representing one classified species) that showed the lowest diversity were selected, and the two sequences' alignment profiles are shown in Figures 1, 2.

#### 3.1.2. Identification of conserved and variable sequences

First, we identified the *Bm86* regions that are conserved at least between the two closest genetic variants belonging to the two closest classified species within *Rhipicephalus* and *Hyalomma* genera to eliminate them when selecting the minimal partial sequences required for species delineation (Figures 1, 2).

Analysis of all possible combinations of two sequence alignments for all selected variants belonging to the *Rhipicephalus* species revealed that only the alignment profile resulting from *Rh. microplus* and *Rh. annulatus* variants showed one conserved region of 786 bp (from 465 to 1,250) and two variable regions. The first variable region is located from position 33 to position 464 by having a size of 432 pb, while the second variable region begins just after the conserved region and continues until the end of the *Bm86* cDNA sequence (from position 1,251 to position 2,054, 804 bp).

Moreover, the same analysis was performed for the selected genetic variants of each *Hyalomma* species. The two sequences' alignments showed that only the alignment between the two selected genetic variants of *Hy. excavatum* and *Hy. anatolicum* showed a conserved region having a size of 455 bp (from 1,092 to 1,546) and two variable regions of 999 bp (from 93 to 1,091) and 367 bp (from 1,547 to 1,913).

#### 3.1.3. Selection of partial sequences required for species delineation

For the two genera and only within these two variable regions, successive phylogenetic analyses were performed using different window lengths by transferring approximately 50 nucleotides from one assay to another. After evaluating several fragment sizes, we found that the minimum size to delineate species in each genus was located for *Rhipicephalus* spp. in the second variable region from the position 1,487 to 1,884 with a size of 398 bp (Figure 1) and in the first variable region from the position 539 to 1,097 with a size of 559 bp for *Hyalomma* species (Figure 2).

### 3.2. Morphological and molecular identification of tick species

Based on the morphological characteristics, collected tick specimens were classified into two genera and eight species: *Hyalomma scupense, Hy. anatolicum, Hy. excavatum, Hy. marginatum, Hy. impeltatum, Hy. dromedarii, Rhipicephalus sanguineus* sensu lato, and *Rh. bursa*. It is relevant to note that this is the first time that *H. anatolicum* has been recorded in Northern Tunisia in the humid zone. Tick species identification was also confirmed by the analysis of mitochondrial 16S rRNA partial sequences (272 bp, GenBank accession numbers OP749949-OP749962). Blast analysis showed 99–100% identity of our sequences isolated from *Hyalomma* and *Rhipicephalus* spp. with those genetically closest published in GenBank, which thus confirms the morphological diagnosis (Table 1).

### 3.3. Amplification of minimal length *Bm86* cDNA partial sequences

Selected tick field samples, nine from *Hyalomma* spp. and five from *Rhipicephalus*, were amplified using the specific set of primers AD-Bm86-Hyl and AR-Bm86-Hyl as well as AD-Bm86-Rhip and AR-Bm86-Rhip with the optimized PCR conditions. All *Hyalomma* samples gave an amplicon of the expected size (603 bp) when amplified with primers specific for *Hyalomma* spp. and reacted negative when amplified with primers specific for *Rhipicephalus* species (Figure 3A). Similarly, all *Rhipicephalus* samples gave an amplicon of the expected size (502 bp) only when amplified using the set of primers specific for *Rhipicephalus* species (Figure 3B). Using the newly designed primers, the cDNA *Bm86* was not amplified for any of the *Rhipicephalus* spp. tick samples using the specific primers for the *Hyalomma* genus, and similarly, cDNA *Bm86* was not amplified for any of the *Hyalomma* spp. tick samples using the specific primers for the *Rhipicephalus* genus. Our results clearly indicate that the newly developed PCR assays were highly specific and capable of accurately distinguishing between closely related tick genera, such as *Hyalomma* and *Rhipicephalus*. Additionally, the sensitivity of the assays was found to be 1.4 pg/μl and 1.9 pg/μl for the genus-specific PCRs for *Hyalomma* and *Rhipicephalus*, respectively (Figure 4).

### 3.4. Genetic analysis of minimal length *Bm86* cDNA partial sequences

To assess the taxonomic interest of *Bm86* orthologs, the amplified fragments of 559 bp and 460 bp containing partial targeted sequences of the *Bm86* transcript were sequenced for *Hyalomma* and *Rhipicephalus* spp., respectively. All sequences were subjected to a Basic Local Alignment Search Tool (BLAST) search to confirm tick species and estimate the homology rate of each sequence with the closest sequence already published in GenBank.

Blast analysis performed on the sequenced *Hy. scupense* ortholog showed 100% sequence homology with the previously published *Hy. scupense* strain Beja (GenBank accession number HQ872022) recovered from cattle in Tunisia. Only one genotype HmBm86G1 was revealed from three *Hy. marginatum* isolates which also shared 100% homology with the *Hy. marginatum* strain Kutahya from Turkey (GenBank accession number KF527438). Two distinct and novel genotypes (HeBm86G1 and HeBm86G1) were identified (GenBank accession numbers OP762546 and OP762546) from two *Hy. excavatum* isolates which shared 99% homology with *Hy. excavatum* strain Sousse recovered from cattle in Tunisia (GenBank accession number JF298786). For *Hy. anatolicum* and *Hy. dromedarii* orthologs, nucleotide sequence identities were superior to 99% in comparison with other previously published *Bm86 Hyalomma* species, resulting in one novel genotype each (HaBm86G1 and HdBm86G1, respectively; Table 1). Since there is no published sequence in GenBank of the *Bm86* gene from *Hy. impeltatum*, one genotype HiBm86G1 was revealed as novel, and blast analysis performed on the *Bm86* ortholog isolated from *Hy. impeltatum* (GenBank accession number OP762551) showed that this sequence has 97.6% sequence homology to the closest sequence that was isolated from the *Hy. anatolicum* isolate Alborz found in an Iranian sheep (GenBank accession number MH325952).

Based on nucleotide alignments of *Bm86* partial nucleotidic sequences (413 bp) of four Tunisian *Rhipicephalus sanguineus sensu lato* isolates, a total of three distinct and novel genotypes RsBm86G1-3 were identified (GenBank accession numbers OP762554 to OP762557). The revealed genotypes were 96.8% to 97.3% similar to *Rh. sanguineus* s.l. (GenBank accession number EF222203; Table 1). Since this is the first time that the *Bm86* ortholog has been sequenced in *Rhipicephalus bursa*, one genotype RbBm86G1 was revealed as novel, and the blast analysis indicated that the closest sequence was that of *Rhipicephalus evertsi evertsi* from South Africa (GU144600) with 96.6% homology (Table 1).

### 3.5. Comparative analysis of phylogenetic trees built for species delineation

Phylogenetic trees based on the alignment of studied minimum length *Bm86* fragments belonging to both *Hyalomma* and *Rhipicephalus* species were compared with those generated from all complete or nearly complete *Bm86* gene sequences of all *Hyalomma* and *Rhipicephalus* species found in GenBank.

**Figure 5:**
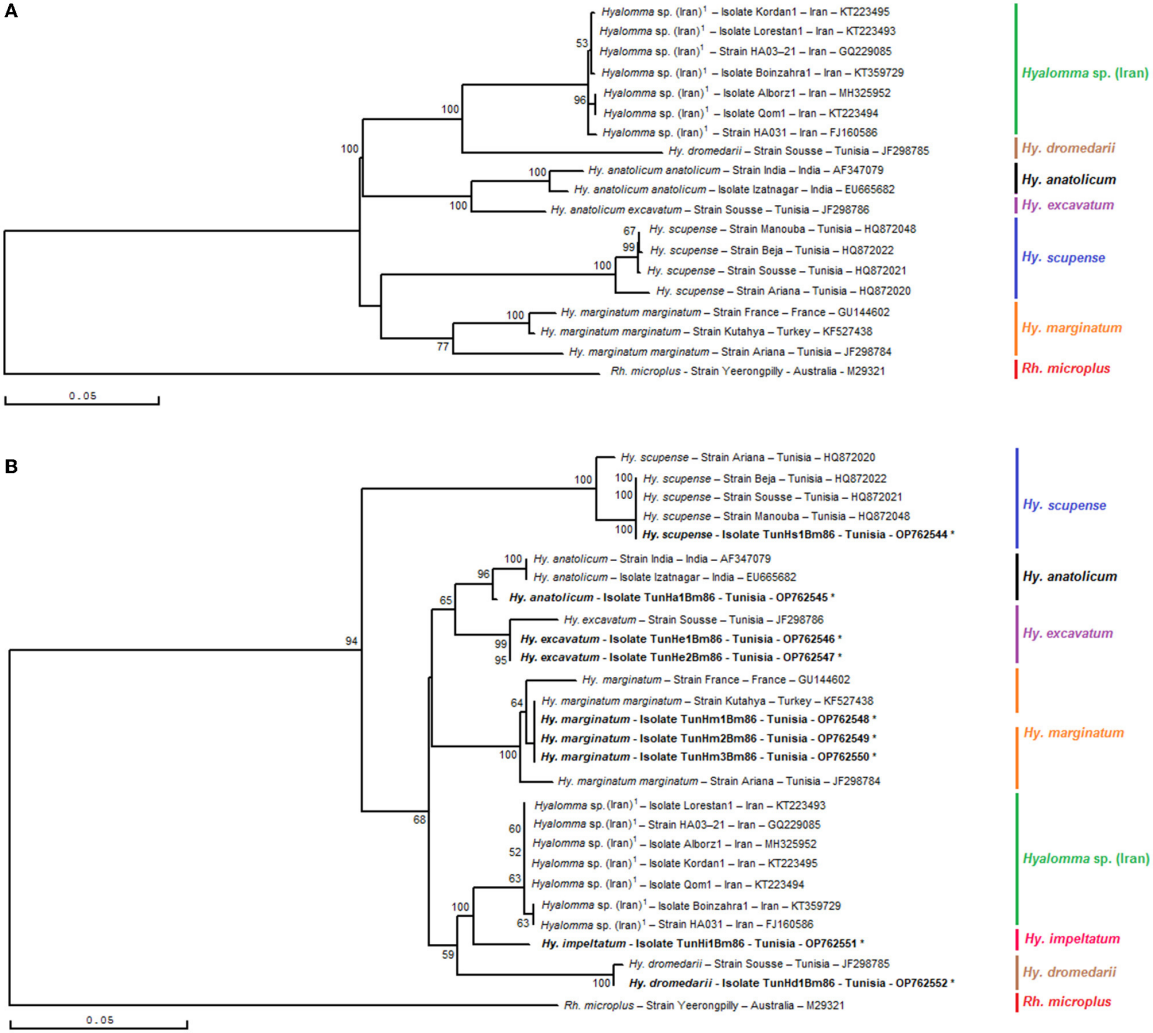
Phylogenetic tree inferred with complete or nearly complete *Bm86* gene sequences **(A)** and selected minimal partial *Bm86* sequences (559 bp) **(B)** required for species delineation within *Hyalomma* tick genera using the neighbor-joining method. Numbers associated with nodes represent the percentage of 1,000 bootstrap iterations supporting the nodes (only percentages >50% are show). The host or vector, strain or isolate name, the country of origin, and the GenBank accession number are indicated. The Yeerongpilly strain of *Rhipicephalus* (*Boophilus*) *microplus* tick from Australia (M29321) was added as an out-group.

Phylogenetic analysis based on the alignment of Tunisian genotypes with different minimal partial sequences of the *Bm86* gene of all classified species of the genus *Hyalomma* obtained from the GenBank generated various clades (Figures 5A, B). The phylogenetic tree demonstrated the discriminatory power of the minimal *Bm86* fragment between *Hyalomma* species. Both revealed *Hy. excavatum* Tunisian strains were classified separately with the Tunisian ortholog strain Sousse isolated from cattle (GenBank accession number JF298786) within *Hy. excavatum* cluster. This latter strain was closely related to the Tunisian *Hy. anatolicum* isolate TunHa1Bm86 found on cattle (GenBank accession number OP762545), which was clustered within the *Hy. anatolicum* cluster containing both orthologs, strain India and isolate Izatnagar, isolated from *Hyalomma anatolicum* in India (GenBank accession numbers AF347079 and EU665682, respectively).

Moreover, the studied minimal length *Bm86* fragment allowed the same isolates' discrimination within each one of the *Hyalomma* species than that obtained with the complete or nearly complete sequences. In particular, as shown by the *Hyalomma* tree generated by complete or nearly complete sequences, all *Hy. marginatum* Tunisian strains were grouped into one *Hy. marginatum* sub-cluster with the Kutahya strain isolated from Turkey and closely related to *Hy. marginatum* strain France and strain Ariana isolated from Tunisian cattle (GenBank accession numbers GU144602 and JF298784, respectively) in the tree based on our minimal length *Bm86* fragment. Furthermore, the studied minimal length *Bm86* fragment allowed the discrimination within *Hy*. *scupense* isolates resembling that made by the complete or nearly complete sequences. In fact, the Tunisian *Hy. scupense* isolate TunHs1Bm86 found on cattle (GenBank accession number OP762544) was grouped into one of two *Hy. scupense* sub-clusters containing three ortholog strains, namely, Beja, Sousse, and Manouba from Tunisia and thereby leaving the *Hy. scupense* strain Ariana from Tunisia (GenBank accession number HQ872020) in a different separate *Hy. scupense* sub-cluster (Figures 5A, B).

**Figure 6:**
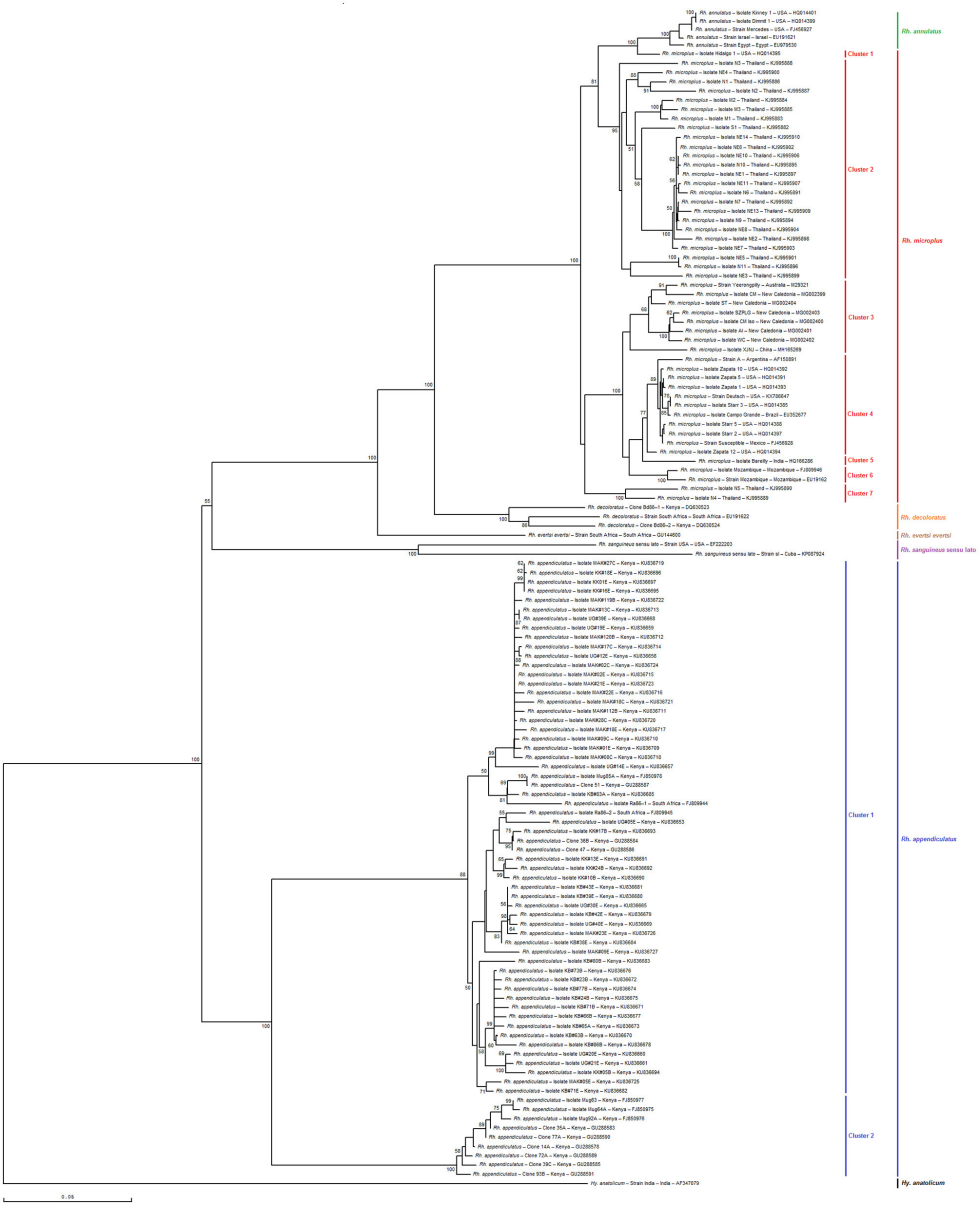
Phylogenetic tree inferred with complete or nearly complete *Bm86* gene sequences required for species delineation within *Rhipicephalus* tick genera using the neighbor-joining method. Numbers associated with nodes represent the percentage of 1,000 bootstrap iterations supporting the nodes (only percentages >50% are show). The host or vector, strain or isolate name, the country of origin, and the GenBank accession number are indicated. The India strain of *Hyalomma anatolicum* tick from India (AF347079) was added as an out-group.

**Figure 7:**
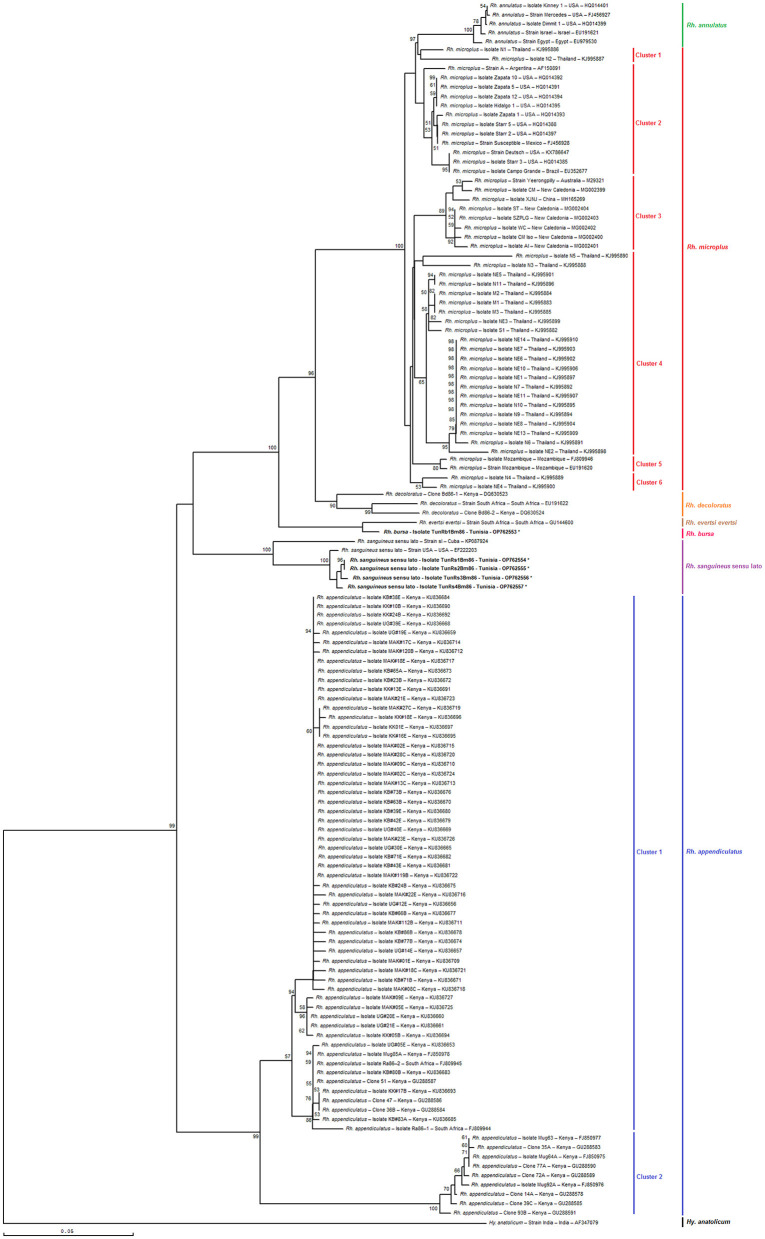
Phylogenetic tree inferred with selected minimal partial *Bm86* gene sequences (398 bp) required for species delineation within *Rhipicephalus* tick genera using the neighbor-joining method. Numbers associated with nodes represent the percentage of 1,000 bootstrap iterations supporting the nodes (only percentages >50% are shown). The host or vector, strain or isolate name, the country of origin, and the GenBank accession number are indicated. The India strain of *Hyalomma anatolicum* tick from India (AF347079) was added as an out-group.

As shown in Figures 6, 7, the phylogenetic tree based on selected minimal partial *Bm86 Rhipicephalus* spp. fragments exhibited almost the same topology found by the analysis of complete or nearly complete *Bm86* gene sequences from all available *Rhipicephalus* species available in GenBank. Almost no changes in phylogenetic relationships were observed. All revealed Tunisian *Rhipicephalus* spp. isolates were classified into the *Rh. sanguineus* s.l. cluster which was closely related to the USA strain (GenBank accession number EF222203) except for the novel Tunisian *Rh. bursa* isolate which was closely related to *Rh. evertsi evertsi* strain from South Africa (GenBank accession number GU144600).

The results also revealed that the minimal length *Bm86* transcript sequence specific to the *Rhipicephalus* genus was effective in discriminating between different groups of *Rh. microplus* and *Rh. appendiculatus* isolates. Specifically, phylogenetic analysis based on the minimal length *Bm86* transcript sequence generated groups of *Rh. microplus* isolates based on the geographic regions similar to those found by the phylogenetic analysis based on the complete or nearly complete sequence. In fact, the groups of isolates from the North and Latin America group (USA, Mexico, and Brazil), Asia-Pacific group (New Caledonia, Australia, China, and India), Thailand, and Mozambique were revealed in both phylogenetic trees based on the two types of sequences (Figures 6, 7). In addition, our partial sequence was able to distinguish between two exceptionally divergent homologs (Ra86) of *Rh. appendiculatus* isolates in a manner similar to the complete or nearly complete transcript *Bm86* sequence, as indicated by the two types of generated trees (Figures 6, 7).

Both phylogenetic trees, constructed by aligning the studied minimum length *Bm86* fragments with complete or nearly complete *Bm86* gene sequences of all *Rhipicephalus* species available in GenBank, clearly showcased the remarkable discriminatory ability of the minimal *Bm86* fragment in distinguishing *Rhipicephalus* species at a phylogeographic level. In particular, as shown by the *Rhipicephalus* tree generated by minimum length *Bm86* fragments, all *Rh. microplus* sequences isolated from Thailand were grouped into *Rh. microplus* clusters 1, 4, and 6, which were also grouped into *Rh. microplus* clusters 2 and 7 regarding the *Rhipicephalus* tree generated by complete or nearly complete *Bm86* gene sequences. Nonetheless, the studied minimal length *Bm86* fragment allowed the discrimination within *Rh. microplus* sequences isolated from countries of the American continent (USA, Mexico, Brazil, and Argentina) which were grouped into *Rh. microplus* cluster 2 and *Rh. microplus* sequences isolated from countries of the African continent, specifically from Mozambique, which were grouped into *Rh. microplus* cluster 5. A consistent discrimination profile was created similar to that made by the *Rhipicephalus* phylogenetic tree generated by complete or nearly complete *Bm86* gene sequences, in which *Rh. microplus* sequences isolated from countries of the American continent were grouped into *Rh. microplus* clusters 1 and 4, and *Rh. microplus* sequences isolated from Mozambique were grouped into *Rh. microplus* cluster 6 (Figures 6, 7).

Furthermore, the studied minimal length *Bm86* fragment allowed the discrimination within *Rh. annulatus* isolates similar to that made by the complete or nearly complete sequences. In particular, five isolates were grouped into *Rh. annulatus* cluster that differ by the country of origin, a total of three *Rh. annulatus* isolates from the USA, one *Rh. annulatus* strain from Palestine (GenBank accession number EU191621), and one *Rh. annulatus* strain from Egypt (GenBank accession number EU979530) (Figures 6, 7).

## 4. Discussion

The identification of tick species has traditionally relied on morphological methods, but these methods can be difficult and subjective. Difficulties may arise during the morphological diagnosis of tick species due to the presence of significant morphological variations related to the stages of development, the state of engorgement of ticks, and the possible presence of hybrid specimens resulting from crossovers between two closely related species (52–54). In recent years, the use of DNA markers for determining tick species has gained widespread popularity due to its precision and efficiency compared to traditional morphological methods (17) and its ability to provide more detailed information about the evolutionary relationships between different species (55).

In recent years, several DNA markers such as the internal transcribed spacer (ITS) region, the cytochrome c oxidase subunit I (COI) gene, and the 16S rRNA gene have been used for tick species identification (15, 52, 56, 57). However, despite their wide use, these markers have been found to have limitations in terms of their discriminatory power and sensitivity (15, 27, 58, 59). For example, the ITS region has been shown to have limited resolution in differentiating between closely related species (27), while the COI gene has been found to have low sensitivity in detecting intraspecific genetic variations (27). Similarly, the 16S rRNA gene has limitations in its ability to differentiate between tick species that belong to different genera (60). In light of these limitations, there is a need for more effective DNA markers that can provide better tick species identification. These limitations are the reason where the proposed study comes in, as it aims to identify the minimum length of partial *Bm86* cDNA fragments needed for species delineation in the tick genera *Rhipicephalus* and *Hyalomma*.

The *Bm86* cDNA marker represents a new and innovative way of species delineation in ticks as it targets the tick's RNA banks that have been previously utilized in various research studies. In many research laboratories, these RNA libraries are for the most part widely studied for their potential in the development of anti-tick vaccines mainly based on proteins from the intestine and salivary glands of ticks (61–65) and in the search for RNA viruses that infect ticks such as tick-borne encephalitis virus (TBEV) and Kyasanur forest disease virus (KFDV) (66–71). In fact, the *Bm86* cDNA marker could represent a significant advance in the field of delineating tick species that make up these banks, providing a sensitive and discriminatory tool that researchers can use in interspecific and intraspecific diversity analyses. Its ability to target the tick's RNA banks, which have already been utilized in previous research, offers new avenues for a more accurate and efficient method for tick species and isolate identification, which will have important implications for ticks and tick-borne disease research and control.

The selection of the *Bm86* transcript as a species identification marker for ticks in the genera *Hyalomma* and *Rhipicephalus* was motivated by several factors. First, this transcript had been previously used in commercial and experimental anti-tick vaccines (48, 49, 72–75), making it a readily available target in tick RNA banks (64, 76). Second, research showed that the phylogeny based on the complete or almost complete sequence of this transcript was in perfect agreement with the recent taxonomy of hard ticks (31, 77). This made it an ideal candidate for species identification as it had already demonstrated its utility in representing the genetic diversity of these ticks (78–81). Furthermore, using this transcript as a marker for species identification would help to better understand the evolution and ecology of these ticks, which are important vectors of various diseases affecting livestock and humans.

However, the purpose of our study was to identify the minimum length partial *Bm86* cDNA fragments required to differentiate between species within the tick genera *Rhipicephalus* and *Hyalomma*. Using a shorter sequence has practical benefits as it only requires one primer pair for amplification and a single primer for sequencing. This reduces the cost of the test compared to analyzing the complete or nearly complete sequence, which requires multiple primer pairs.

Through a combination of genetic analysis and phylogenetic analysis, the study identified the conserved and variable regions within the *Bm86* cDNA of these tick species. The crucial factor in choosing the partial sequences specific to *Hyalomma* and *Rhipicephalus* genera was the alignment of two genetically closest species *Hy. excavatum* and *Hy. anatolicum* (96.9% identity rate) and *Rh. microplus* and *Rh. annulatus* (98.6%), respectively. As a result of these alignments, one conserved and two variable regions were identified in each genus, as shown in Figure 2 for *Hyalomma* and Figure 1 for *Rhipicephalus*. Therefore, to ensure accurate results in future molecular diagnosis studies of *Hyalomma* and *Rhipicephalus* spp., we suggest avoiding the use of partial sequences within these conserved regions and instead focusing on amplifying and sequencing fragments found in the two variable regions of each genus (Figures 1, 2). By evaluating different fragment sizes within the variable regions, we determined that the minimum size for species delineation was 398 bp for *Rhipicephalus* spp. (from 1,487 to 1,884) and 559 bp for *Hyalomma* species (from 539 to 1,097).

The study also confirmed tick species identification through morphological characteristics and mitochondrial 16S rRNA partial sequences, with a 99.27–100% identity with the closest published *Hyalomma* and *Rhipicephalus* sequences in GenBank. Interestingly, we identified a *Hyalomma anatolicum* tick specimen based on its morphological characteristics, and its identity was validated by mitochondrial 16S rRNA partial sequences. The validation revealed a 100% match with the closest published *Hyalomma anatolicum* sequence in GenBank. Notably, this finding marks the second report of this tick species since the discovery of a single *Hyalomma anatolicum* specimen in 1970 by Van Den Ende in Southern Tunisia (82). This discovery highlights the risk that this tick becomes a vector of the apicomplexan hemoparasite *Theileria annulata* in Tunisia as already advanced by Gharbi et al. (83).

Furthermore, the field evaluation study successfully amplified and genetically analyzed the minimal length *Bm86* cDNA partial sequences for the identified tick species, with 99.10–100% and 96.85–97.34% homology rates with previously published *Hyalomma* and *Rhipicephalus* sequences, respectively. These findings have important implications for the accurate identification and classification of tick species, which is crucial for understanding the epidemiology and transmission of tick-borne diseases.

In addition, our results also support the utility of *Bm86* orthologs as a potential marker for tick isolate or strain identification. In particular, the results suggest that the studied minimal length *Bm86* fragment is just as effective at discriminating between isolates and strains within each *Hyalomma* and *Rhipicephalus* species as the complete or nearly complete sequences. The phylogenetic inference based on the two partial sequences that were chosen showed the same pattern as previously observed when using the complete or nearly complete sequence (Figures 5, 6). No changes in the relationships between the isolates and strains of each species were detected, which suggests that there is no incongruity in the phylogeny. The trees generated using the neighbor-joining method had high support values at the terminal nodes and most of the deeper branches. There were also no instances of polytomies, which is likely because the selected fragments contained the most informative sites, resulting in a conservation of the phylogenetic signal. These findings were consistent with previous studies (84, 85). This is interesting because it suggests that this shorter fragment could be a more efficient and cost-effective reliable method for identifying and characterizing *Hyalomma* isolates and strains. In fact, our findings indicate that the two *Hyalomma* trees generated using the complete or nearly complete sequences and the minimal sequence showed similar results. Specifically, the genetic analysis revealed that the *Hy. marginatum* strain from Turkey (Kutahya) (86) is more closely related to the *Hy. marginatum* strain from France (80) than to all the *Hy. marginatum* Tunisian strains (31). Additionally, the isolate Ariana from *Hy. scupense* was relatively more distant from the isolates from Sousse, Manouba, and Beja (31).

Additionally, our result suggests that the minimal length *Bm86* transcript sequence selected in this study can be as effective as the complete or nearly complete sequence in discriminating between isolates and strains within *Rh. microplus* and *Rh. appendiculatus* species. First, the phylogenetic analysis based on the minimal length sequence was able to generate groups of isolates based on their geographic regions, which were similar to the groups generated by the analysis based on the complete or nearly complete sequence. Second, the results showed that the minimal length *Bm86* transcript sequence specific to the *Rhipicephalus* genus was also effective in discriminating between the two genospecies groups of *Rh. appendiculatus* isolates (87) in a similar way than with the complete or nearly complete transcript *Bm86* sequence (Figures 6, 7). This finding indicates that the shorter fragment could also be a useful tool for identifying and characterizing *Rhipicephalus* strains. Overall, these results highlight the potential benefits of using shorter sequence fragments to identify and distinguish between different strains and isolates of tick species belonging to these two genera, which could have important implications for tick control and disease prevention strategies. However, it is important to note that further research may be necessary to confirm these findings and to determine whether they are applicable to other tick species.

However, the primers we designed for the amplification of each *Bm86* minimum partial sequence specific to *Hyalomma* and *Rhipicephalus* were found to be sensitive and specific for each targeted tick genus. We were able to confirm this by successfully obtaining an amplicon of the expected size for all samples when amplified with primers specific for their respective genus. This indicates that the two sets of primers we created are suitable for amplifying and sequencing our cDNA marker and are capable of differentiating between the two closely related tick genera.

The use of a minimal *Bm86* cDNA sequence for tick species differentiation offers advantages such as the use of a single pair of genus-specific primers for amplification and only a single primer for sequencing, followed by a simple blast analysis to determine the species within each genus. However, it is important to acknowledge the limitations associated with cDNA, as researchers need to extract total RNA and obtain cDNA through reverse transcription. Nonetheless, the decision to exclusively use cDNA for both the *Hyalomma* and *Rhipicephalus* genera was made due to the presence of a large 2,888 bp intron at position 1,752 bp in *Bm86* cDNA (88), which is located within the minimal sequence used to discriminate *Rhipicephalus* species. Consequently, amplification of gDNA using specific primers for the *Rhipicephalus* genus is not feasible. To maintain consistency, we chose to utilize cDNA exclusively for both *Hyalomma* and *Rhipicephalus* genera.

Furthermore, this cDNA marker is expected to be particularly useful in identifying tick specimens that are difficult to recognize morphologically, for instance, with fully engorged tick immature females, in case of occurrence of malformations, or in the occurrence of hybrids of two species of ticks that are taxonomically closely related (31, 89, 90). Our marker, which is able to accurately identify tick species that may be otherwise difficult to differentiate, could be used as an additional taxonomic tool for morphological diagnosis for improving the reliability of tick species identification, particularly when considering the increased risks for changes in tick fauna and the emergence of new tick species driven by the combination of climate change with anthropogenic factors.

## 5. Conclusion

Genomic and mitochondrial DNA markers have been shown to be effective in identifying tick species compared to traditional morphological methods. In addition to these existing markers, we have identified here a novel *Bm86* cDNA marker that provides a new approach to tick species identification and can target tick RNA banks. In this study, we developed a molecular method based on the minimum length of partial *Bm86* cDNA fragments needed for identifying accurately tick species within the *Rhipicephalus* and *Hyalomma* genera, increasing, therefore, the range of taxonomic tools applied to identify reliably hard tick species.

## Data availability statement

The datasets presented in this study can be found in online repositories. The names of the repository/repositories and accession number(s) can be found in the article/Supplementary material.

## Ethics statement

The collection of ticks from cattle was done in accordance with the Tunisian National School of Veterinary Medicine's guidelines for animal care and handling and required no ethical approval. Consent was sought from the farmers before sampling. The animals were gently restrained by their owners in the same way as during routine clinical examinations, and invasive sampling or tranquilizers were not used. Moreover, the sampling was overseen by veterinarians and veterinary technicians from the National School of Veterinary Medicine in Sidi Thabet.

## Author contributions

MBS conceived the idea, conducted *in silico* analysis, and designed the experiments. SZ, MM, and MD performed the experiments. SZ and MBS wrote the manuscript. MAD collaborated with MBS in developing the study concept and in editing and finalizing the manuscript. All authors have reviewed and approved the current version of the manuscript.
