# Supplementary material for: Development and field evaluation of PCR assays based on minimum length Bm86 cDNA fragments required for Rhipicephalus and Hyalomma tick species delineation
Source: Front Vet Sci. 2023 Jun 29;10:1209210. doi: 10.3389/fvets.2023.1209210 (PMC10340088; doi:10.3389/fvets.2023.1209210)
Supplement: Supplementary file 1 [file Table_1.DOCX]

**TABLE S1** Information about fully and nearly complete *Bm*86 cDNA isolated from *Rhipicephalus* *microplus* and *Rh. annulatus* used for the selection of minimum length partial sequence required for *Rhipicephalus* species attribution.

| *Rhipicephalus* spp. | Strain or Isolate | Country | GenBank^1^ | Query recovery (%) | Identity rate (%) | Reference |
| --- | --- | --- | --- | --- | --- | --- |
| *Rh. microplus* | Strain Yeerongpilly | Australia | M29321 | 100 | 100 | (60) |
|  | Strain A | Argentina | AF150891 | 100 | 98.38 | (40) |
|  | Isolate Rmic-2018 | China | XM037422513 | 99 | 96.95 | *Unpublished* |
|  | Isolate Mozambique | Mozambique | FJ809946 | 95 | 97.23 | (41) |
|  | Isolate Zapata 10 | USA | HQ014392 | 90 | 98.12 | (42) |
|  | Isolate Zapata 5 | USA | HQ014391 | 90 | 98.12 |  |
|  | Isolate Zapata 1 | USA | HQ014393 | 90 | 98.07 |  |
|  | Isolate Starr 5 | USA | HQ014388 | 90 | 98.07 |  |
|  | Isolate Starr 2 | USA | HQ014397 | 90 | 98.02 |  |
|  | Isolate Zapata 12 | USA | HQ014394 | 90 | 98.02 |  |
|  | Strain Deutsch | USA | KX786647 | 90 | 97.92 | *Unpublished* |
|  | Isolate Starr 3 | USA | HQ014385 | 90 | 97.92 | (42) |
|  | Isolate Zapata 11 | USA | HQ014398 | 90 | 97.77 |  |
|  | Isolate Starr 1 | USA | HQ014396 | 90 | 97.58 |  |
|  | Isolate XJNJ | China | MH165269 | 87 | 98.62 | (80) |
|  | Haplotype MxHT6 | Mexico | MG988331 | 87 | 98.31 | *Unpublished* |
|  | Haplotype MxHT31 | Mexico | MG988356 | 87 | 98.11 |  |
|  | Haplotype MxHT13 | Mexico | MG988338 | 87 | 98.11 |  |
|  | Haplotype MxHT5 | Mexico | MG988330 | 87 | 98.11 |  |
|  | Haplotype MxHG3 | Mexico | MG988319 | 87 | 98.11 |  |
|  | Haplotype MxHG2 | Mexico | MG988318 | 87 | 98.11 |  |
|  | Haplotype MxHT52 | Mexico | MG988377 | 87 | 98.05 |  |
|  | Haplotype MxHT15 | Mexico | MG988340 | 87 | 98.05 |  |
|  | Haplotype MxHT8 | Mexico | MG988333 | 87 | 98.05 |  |
|  | Haplotype MxHG7 | Mexico | MG988323 | 87 | 98.05 |  |
|  | Haplotype MxHT30 | Mexico | MG988355 | 87 | 98.00 |  |
|  | Haplotype MxHT17 | Mexico | MG988342 | 87 | 98.00 |  |
|  | Haplotype MxHT16 | Mexico | MG988341 | 87 | 98.00 |  |
|  | Haplotype MxHG6 | Mexico | MG988322 | 87 | 98.00 |  |
|  | Haplotype MxHT53 | Mexico | MG988378 | 87 | 97.95 |  |
|  | Haplotype MxHT19 | Mexico | MG988344 | 87 | 97.95 |  |
|  | Haplotype MxHT12 | Mexico | MG988337 | 87 | 97.95 |  |
|  | Haplotype MxHT1 | Mexico | MG988317 | 87 | 97.95 |  |
|  | Haplotype MxHT28 | Mexico | MG988353 | 87 | 97.90 |  |
|  | Haplotype MxHT25 | Mexico | MG988350 | 87 | 97.90 |  |
|  | Haplotype MxHT10 | Mexico | MG988335 | 87 | 97.90 |  |

**TABLE S1** *Continued*

| *Rhipicephalus* spp. | Strain or Isolate | Country | GenBank^2^ | Query recovery (%) | Identity rate (%) | Reference |
| --- | --- | --- | --- | --- | --- | --- |
| *Rh. microplus* | Haplotype MxHT9 | Mexico | MG988334 | 87 | 97.90 | *Unpublished* |
|  | Haplotype MxHT7 | Mexico | MG988332 | 87 | 97.90 |  |
|  | Haplotype MxHG4 | Mexico | MG988320 | 87 | 97.90 |  |
|  | Haplotype MxHT21 | Mexico | MG988346 | 87 | 97.85 |  |
|  | Haplotype MxHT18 | Mexico | MG988343 | 87 | 97.85 |  |
|  | Haplotype MxHT11 | Mexico | MG988336 | 87 | 97.85 |  |
|  | Haplotype MxHT4 | Mexico | MG988329 | 87 | 97.85 |  |
|  | Haplotype MxHT2 | Mexico | MG988327 | 87 | 97.85 |  |
|  | Haplotype MxHT51 | Mexico | MG988376 | 87 | 97.80 |  |
|  | Haplotype MxHT42 | Mexico | MG988367 | 87 | 97.80 |  |
|  | Haplotype MxHT41 | Mexico | MG988366 | 87 | 97.80 |  |
|  | Haplotype MxHT22 | Mexico | MG988347 | 87 | 97.80 |  |
|  | Haplotype MxHT14 | Mexico | MG988339 | 87 | 97.80 |  |
|  | Haplotype MxHT3 | Mexico | MG988328 | 87 | 97.80 |  |
|  | Haplotype MxHT1 | Mexico | MG988326 | 87 | 97.80 |  |
|  | Haplotype MxHT34 | Mexico | MG988359 | 87 | 97.75 |  |
|  | Haplotype MxHT33 | Mexico | MG988358 | 87 | 97.75 |  |
|  | Haplotype MxHT27 | Mexico | MG988352 | 87 | 97.75 |  |
|  | Haplotype MxHT26 | Mexico | MG988351 | 87 | 97.75 |  |
|  | Haplotype MxHG8 | Mexico | MG988324 | 87 | 97.75 |  |
|  | Haplotype MxHT35 | Mexico | MG988360 | 87 | 97.70 |  |
|  | Haplotype MxHT23 | Mexico | MG988348 | 87 | 97.70 |  |
|  | Haplotype MxHT50 | Mexico | MG988375 | 87 | 97.64 |  |
|  | Haplotype MxHT20 | Mexico | MG988345 | 87 | 97.64 |  |
|  | Haplotype MxHT36 | Mexico | MG988361 | 87 | 97.59 |  |
|  | Haplotype MxHT32 | Mexico | MG988357 | 87 | 97.59 |  |
|  | Haplotype MxHT43 | Mexico | MG988368 | 87 | 97.54 |  |
|  | Haplotype MxHT49 | Mexico | MG988374 | 87 | 97.49 |  |
|  | Haplotype MxHT48 | Mexico | MG988373 | 87 | 97.49 |  |
|  | Haplotype MxHT47 | Mexico | MG988372 | 87 | 97.49 |  |
|  | Haplotype MxHT40 | Mexico | MG988365 | 87 | 97.49 |  |
|  | Haplotype MxHT46 | Mexico | MG988371 | 87 | 97.44 |  |
|  | Haplotype MxHT45 | Mexico | MG988370 | 87 | 97.39 |  |
|  | Haplotype MxHT24 | Mexico | MG988349 | 87 | 97.34 |  |
|  | Isolate ST | New Caledonia | MG002404 | 82 | 99.29 | (81) |
|  | Isolate CM | New Caledonia | MG002399 | 82 | 99.23 |  |

**TABLE S1** *Continued*

| *Rhipicephalus* spp. | Strain or Isolate | Country | GenBank^2^ | Query recovery (%) | Identity rate (%) | Reference |
| --- | --- | --- | --- | --- | --- | --- |
| *Rh. microplus* | Haplotype MxHT37 | Mexico | MG988362 | 87 | 97.03 | *Unpublished* |
|  | Haplotype MxHG9 | Mexico | MG988325 | 87 | 96.98 |  |
|  | Haplotype MxHT55 | Mexico | MG988380 | 87 | 96.83 |  |
|  | Isolate SZPLG | New Caledonia | MG002403 | 82 | 98.90 | (81) |
|  | Isolate WC | New Caledonia | MG002402 | 82 | 98.90 |  |
|  | Haplotype MxHT44 | Mexico | MG988369 | 87 | 96.77 | *Unpublished* |
|  | Isolate AI | New Caledonia | MG002401 | 82 | 98.80 |  |
|  | Isolate Hidalgo 1 | USA | HQ014395 | 90 | 95.70 | (42) |
|  | Haplotype MxHT54 | Mexico | MG988379 | 87 | 96.31 | *Unpublished* |
|  | Haplotype MxHT38 | Mexico | MG988363 | 87 | 96.31 |  |
|  | Isolate N5 | Thailand | KJ995890 | 87 | 96.16 | (82) |
|  | Isolate N4 | Thailand | KJ995889 | 87 | 96.16 |  |
|  | Haplotype MxHT39 | Mexico | MG988364 | 87 | 96.16 | *Unpublished* |
|  | Isolate N3 | Thailand | KJ995888 | 87 | 96.06 |  |
|  | Strain Susceptible | Mexico | FJ456928 | 82 | 98.03 | (83) |
|  | Isolate NE4 | Thailand | KJ995900 | 87 | 95.85 | (82) |
|  | Isolate Campo Grande | Brazil | EU352677 | 82 | 97.81 | (85) |
|  | Isolate NE5 | Thailand | KJ995901 | 87 | 95.65 | (82) |
|  | Haplotype MxHT29 | Mexico | MG988354 | 87 | 95.65 | *Unpublished* |
|  | Isolate N11 | Thailand | KJ995896 | 87 | 95.60 | (82) |
|  | Isolate M2 | Thailand | KJ995884 | 87 | 95.60 |  |
|  | Isolate M1 | Thailand | KJ995883 | 87 | 95.60 |  |
|  | Haplotype MxHG5 | Mexico | MG988321 | 87 | 95.55 | *Unpublished* |
|  | Isolate M3 | Thailand | KJ995885 | 87 | 95.49 | (82) |
|  | Isolate S1 | Thailand | KJ995882 | 87 | 95.39 |  |
|  | Isolate N1 | Thailand | KJ995886 | 87 | 95.29 |  |
|  | Isolate NE3 | Thailand | KJ995899 | 87 | 95.14 | (82) |
|  | Strain IVRI-I | India | MK728951 | 87 | 95.08 | *Unpublished* |
|  | Isolate NE14 | Thailand | KJ995910 | 87 | 94.98 | (82) |
|  | Isolate NE7 | Thailand | KJ995903 | 87 | 94.93 |  |
|  | Isolate NE6 | Thailand | KJ995902 | 87 | 94.93 |  |
|  | Isolate NE10 | Thailand | KJ995906 | 87 | 94.88 |  |
|  | Isolate NE1 | Thailand | KJ995897 | 87 | 94.88 |  |
|  | Isolate N7 | Thailand | KJ995892 | 87 | 94.88 |  |
|  | Isolate NE11 | Thailand | KJ995907 | 87 | 94.83 |  |
|  | Isolate N10 | Thailand | KJ995895 | 87 | 94.83 |  |
|  | Isolate N9 | Thailand | KJ995894 | 87 | 94.83 |  |
|  | Isolate NE8 | Thailand | KJ995904 | 87 | 94.78 |  |

**TABLE S1** *Continued*

| *Rhipicephalus* spp. | Strain or Isolate | | Country | GenBank^2^ | Query recovery (%) | Identity rate (%) | Reference |
| --- | --- | --- | --- | --- | --- | --- | --- |
| *Rh. microplus* | Isolate NE2 | Thailand | | KJ995898 | 87 | 94.73 | (82) |
|  | Isolate N6 | Thailand | | KJ995891 | 87 | 94.67 |  |
|  | Isolate N2 | Thailand | | KJ995887 | 87 | 94.67 |  |
|  | Isolate NE13 | Thailand | | KJ995909 | 87 | 94.57 |  |
|  | Isolate Bareilly | India | | HQ166286 | 76 | 98.30 | *Unpublished* |
|  | Strain Mozambique | Mozambique | | EU191620 | 79 | 97.71 | *(84)* |
|  | Isolate CM Iso | New Caledonia | | MG002400 | 75 | 98.90 | (81) |
| *Rh. annulatus* | Isolate Kinney 1 | | USA | HQ014401 | 100 | 100 | (42) |
|  | Isolate Dimmit 1 | | USA | HQ014399 | 100 | 99.90 |  |
|  | Strain Mercedes | | USA | FJ456927 | 90 | 99.29 | (83) |
|  | Strain Egypt | | Egypt | EU979530 | 96 | 96.97 | (86) |
|  | Strain Israel | | Israel | EU191621 | 90 | 97.59 | (84) |

**TABLE S2** Information about fully and nearly complete *Bm*86 cDNA isolated from *Rhipicephalus* *appendiculatus, Rh. sanguineus* sensu lato*, Rh. evertsi evertsi* and *Rh. decoloratus* used for the selection of minimum length partial sequence required for *Rhipicephalus* species attribution.

| *Rhipicephalus* spp. | Isolate | Country | GenBank^1^ | Query recovery (%) | Identity rate (%) | Reference |
| --- | --- | --- | --- | --- | --- | --- |
| *Rh. appendiculatus* | Isolate Mug85A | Kenya | FJ850978 | 100 | 100 | (77) |
|  | Isolate Ra86-1 | South Africa | FJ809944 | 97 | 98.39 | (41) |
|  | Isolate Ra86-2 | South Africa | FJ809945 | 97 | 98.20 |  |
|  | Isolate KB#83A | Kenya | KU836685 | 80 | 99.33 | (87) |
|  | Isolate MAK#21E | Kenya | KU836723 | 80 | 98.50 |  |
|  | Isolate MAK#02E | Kenya | KU836715 | 80 | 98.50 |  |
|  | Isolate MAK#28C | Kenya | KU836720 | 80 | 98.44 |  |
|  | Isolate MAK#09C | Kenya | KU836710 | 80 | 98.44 |  |
|  | Isolate MAK#02C | Kenya | KU836724 | 80 | 98.39 |  |
|  | Isolate MAK#13C | Kenya | KU836713 | 80 | 98.39 |  |
|  | Isolate UG#39E | Kenya | KU836668 | 80 | 98.39 |  |
|  | Isolate UG#19E | Kenya | KU836659 | 80 | 98.39 |  |
|  | Isolate MAK#119B | Kenya | KU836722 | 80 | 98.33 |  |
|  | Isolate MAK#17C | Kenya | KU836714 | 80 | 98.33 |  |

**TABLE S2** *Continued*

| *Rhipicephalus* spp. | Strain, Isolate or clone | Country | GenBank^2^ | Query recovery (%) | Identity rate (%) | Reference |
| --- | --- | --- | --- | --- | --- | --- |
|  | Isolate MAK#120B | Kenya | KU836712 | 80 | 98.33 |  |
|  | Isolate MAK#01E | Kenya | KU836709 | 80 | 98.33 |  |
|  | Isolate UG#12E | Kenya | KU836656 | 80 | 98.33 |  |
|  | Isolate MAK#27C | Kenya | KU836719 | 80 | 98.28 |  |
|  | Isolate MAK#08C | Kenya | KU836718 | 80 | 98.28 |  |
|  | Isolate MAK#22E | Kenya | KU836716 | 80 | 98.28 |  |
|  | Isolate MAK#112B | Kenya | KU836711 | 80 | 98.28 |  |
|  | Isolate KK01E | Kenya | KU836697 | 80 | 98.28 |  |
|  | Isolate KK#16E | Kenya | KU836695 | 80 | 98.28 |  |
|  | Isolate MAK#18E | Kenya | KU836717 | 80 | 98.22 |  |
|  | Isolate KK#18E | Kenya | KU836696 | 80 | 98.22 |  |
|  | Isolate MAK#18C | Kenya | KU836721 | 80 | 98.11 |  |
|  | Isolate MAK#09E | Kenya | KU836727 | 80 | 98.00 |  |
|  | Isolate KB#80B | Kenya | KU836683 | 80 | 97.94 |  |
|  | Isolate KB#73B | Kenya | KU836676 | 80 | 97.78 |  |
|  | Isolate KB#63B | Kenya | KU836670 | 80 | 97.78 |  |
|  | Isolate MAK#05E | Kenya | KU836725 | 80 | 97.72 |  |
|  | Isolate KK#17B | Kenya | KU836693 | 80 | 97.72 |  |
|  | Isolate KB#43E | Kenya | KU836681 | 80 | 97.72 |  |
|  | Isolate KB#39E | Kenya | KU836680 | 80 | 97.72 |  |
|  | Clone 36B | Kenya | GU288584 | 69 | 97.91 |  |
|  | Isolate Mug63 | Kenya | FJ850977 | 87 | 88.80 |  |
|  | Isolate Mug92A | Kenya | FJ850976 | 87 | 88.80 |  |
|  | Isolate Mug64A | Kenya | FJ850975 | 99 | 88.65 |  |
|  | Isolate UG#14E | Kenya | KU836657 | 69 | 98.72 | (87) |
|  | Isolate KB#38E | Kenya | KU836684 | 75 | 98.21 |  |
|  | Isolate UG#05E | Kenya | KU836653 | 66 | 96.80 |  |
|  | Clone 93B | Kenya | GU288591 | 68 | 90.79 | (77) |
|  | Clone 72A | Kenya | GU288589 | 68 | 90.79 |  |
|  | Clone 39C | Kenya | GU288585 | 68 | 90.64 |  |
|  | Clone 14A | Kenya | GU288578 | 68 | 90.65 |  |
|  | Clone 77A | Kenya | GU288590 | 68 | 90.58 |  |
|  | Clone 35A | Kenya | GU288583 | 68 | 90.51 |  |
| *Rh. sanguineus* sensu lato | Strain USA | USA | EF222203 | 100 | 100 | *Unpublished* |
|  | Strain sl | Cuba | KP087924 | 81 | 93.91 |  |
| *Rh. evertsi evertsi* | Strain South Africa | South Africa | GU144600 | 100 | 100 | (70) |
| *Rh.* *decoloratus* | Clone Bd86-1 | Kenya | DQ630523 | 100 | 100 | (64) |
|  | Strain South Africa | South Africa | EU191622 | 99 | 96.42 | (84) |
|  | Clone Bd86-2 | Kenya | DQ630524 | 100 | 95.94 | (64) |

**TABLE S3** Information about fully and nearly complete *Bm*86 cDNA isolated from available *Hyalomma* species used for the selection of minimum length partial sequence needed for *Hyalomma* species delineation.

| *Hyalomma* spp. | Strain or isolate | Country | GenBank^1^ | Query recovery (%) | Identity rate (%) | Reference |
| --- | --- | --- | --- | --- | --- | --- |
| *Hy. anatolicum anatolicum* | Strain India | India | AF347079 | 100 | 100 | (63) |
|  | Isolate Izatnagar | India | EU665682 | 89 | 99.34 | (88) |
|  | Isolate Lorestan | Iran | KT223493 | 99 | 94.23 | *Unpublished* |
|  | Strain HA03-2 | Iran | GQ229085 | 99 | 94.23 |  |
|  | Isolate Alborz | Iran | MH325952 | 99 | 94.18 |  |
|  | Isolate Boinzahra | Iran | KT359729 | 99 | 94.18 |  |
|  | Isolate Kordan | Iran | KT223495 | 99 | 94.18 |  |
|  | Isolate Qom | Iran | KT223494 | 99 | 94.18 |  |
|  | Strain HA03 | Iran | FJ160586 | 99 | 94.09 |  |
|  | Strain HA03-1 | Iran | GQ228820 | 90 | 94.14 | (89) |
| *Hy. anatolicum excavatum* | Strain Sousse | Tunisia | JF298786 | 100 | 100 | (31) |
| *Hy. marginatum marginatum* | Strain France | France | GU144602 | 100 | 100 | (70) |
|  | Strain Kutahya | Turkey | KF527438 | 92 | 99.57 | (76) |
|  | Strain Ariana | Tunisia | JF298784 | 92 | 97.23 | (31) |
| *Hy. dromedarii* | Strain Sousse | Tunisia | JF298785 | 100 | 100 |  |
| *Hy. scupense* | Strain Manouba | Tunisia | HQ872048 | 100 | 100 |  |
|  | Strain Beja | Tunisia | HQ872022 | 100 | 99.95 |  |
|  | Strain Sousse | Tunisia | HQ872021 | 100 | 99.89 |  |
|  | Strain Ariana | Tunisia | HQ872020 | 98 | 99.18 |  |
